# The anterior midcingulate cortex as a neural node underlying hostility in young adults

**DOI:** 10.1007/s00429-016-1200-6

**Published:** 2016-02-20

**Authors:** Seishu Nakagawa, Hikaru Takeuchi, Yasuyuki Taki, Rui Nouchi, Atsushi Sekiguchi, Yuka Kotozaki, Carlos Makoto Miyauchi, Kunio Iizuka, Ryoichi Yokoyama, Takamitsu Shinada, Yuki Yamamoto, Sugiko Hanawa, Tsuyoshi Araki, Hiroshi Hashizume, Keiko Kunitoki, Yuko Sassa, Ryuta Kawashima

**Affiliations:** 1 Department of Functional Brain Imaging, Institute of Development, Aging and Cancer, Tohoku University, 4-1 Seiryo-machi, Aoba-ku, Sendai, 980-8575 Japan; 2 Department of Psychiatry, Tohoku Pharmaceutical University, Sendai, Japan; 3 Division of Developmental Cognitive Neuroscience, Institute of Development, Aging and Cancer, Tohoku University, Sendai, Japan; 4 Division of Medical Neuroimaging Analysis, Department of Community Medical Supports, Tohoku Medical Megabank Organization, Tohoku University, Sendai, Japan; 5 Department of Nuclear Medicine and Radiology, Institute of Development, Aging and Cancer, Tohoku University, Sendai, Japan; 6 Human and Social Response Research Division, International Research Institute of Disaster Science, Tohoku University, Sendai, Japan; 7 Smart Ageing International Research Center, Institute of Development, Aging and Cancer, Tohoku University, Sendai, Japan; 8 Department of Adult Mental Health, National Institute of Mental Health, National Center of Neurology and Psychiatry, Kodaira, Tokyo Japan; 9 Department of General Systems Studies, Graduate School of Arts and Sciences, The University of Tokyo, Tokyo, Japan; 10 Department of Psychiatry, Tohoku University Graduate School of Medicine, Sendai, Japan; 11 Japan Society for the Promotion of Science, Tokyo, Japan; 12 Faculty of Medicine, Tohoku University, Sendai, Japan

**Keywords:** Anger-Out, Anger-Trait, Hostile behaviors subscale (HBS), Regional gray matter density (rGMD)

## Abstract

Anger typically manifests for only a short period of time, whereas hostility is present for a longer duration. However, both of these emotions are associated with an increased likelihood of psychological problems. The nodes within the neural networks that underlie hostility remain unclear. We presumed that specific nodes might include the anterior midcingulate cortex (aMCC), which seems to be essential for the cognitive aspects of hostility. Thus, the present study first evaluated the associations between regional gray matter density (rGMD) and hostility in 777 healthy young students (433 men and 344 women; 20.7 ± 1.8 years of age) using magnetic resonance imaging and the hostile behaviors subscale (HBS) of the Coronary-prone Type Scale (CTS) for Japanese populations. The HBS scores were positively correlated with rGMD in the aMCC and in widespread frontal regions from the dorsomedial/dorsolateral prefrontal cortices to the lateral premotor cortex at the whole-brain level. No significant correlation was observed between rGMD and the conjunction of HBS and Trait Anger/Anger-Out scores. Furthermore, no significant interaction effects of sex and HBS scores on rGMD were revealed, although the HBS scores of males were significantly higher than those of females. The present findings indicate that the neural correlates of hostility appear to be more distinct in rGMD than those of anger due to differences and duration.

**Electronic supplementary material:**

The online version of this article (doi:10.1007/s00429-016-1200-6) contains supplementary material, which is available to authorized users.

## Introduction

Hostility can be defined as a tendency to feel anger toward and a desire to inflict harm upon a person or group according to the 10th version of the International Statistical Classification of Diseases and Related Health Problems (World Health Organization (2016). Anger is a momentary or passing experience, whereas hostility is not an evanescent experience (Jackson 1972). Moreover, hostility accompanies many other emotional states and pathological conditions (Jackson 1972). Thus, although hostility and anger may overlap to some degree, hostility also likely constitutes a long-acting independent construct entailing specific affective, behavioral and cognitive dimensions (Cox and Harrison 2008).

We should focus on an important aspect of hostility, which is that hostility can lead to negative emotions during interpersonal interactions (Lemerise and Dodge 2008). Hostility is a negative attitude toward others, consisting of enmity, denigration, and ill will (Smith et al. 2004). These negative attitudes can lead to interpersonal rejection, and to the development of critical and relatively severe attitudes (Houston and Vavak 1991). Furthermore, these negative attitudes might facilitate hostile or even aggressive responses (Chen et al. 2012), mainly directed at the destruction of objects, as well as insults or harmful deeds (Ramírez and Andreu 2006). Thus, people prone to hostility will be predisposed to predict negative responses in future interpersonal interactions.

From a clinical perspective, hostility is one of the main symptoms associated with the need for mental healthcare. Hostility is associated with heightened psychosocial vulnerability under conditions of poor psychosocial resources, as well as with an inability to benefit from existing psychosocial resources (Vahtera et al. 2000). Furthermore, hostility was detected in 40.9 % of inpatients in a psychiatric care unit (Raja and Azzoni 2005), and psychiatric nurses in a forensic ward observed that hostile behaviors hindered the therapeutic relationships of patients (Tema et al. 2011).

No studies have investigated the brain structures that support hostility using direct brain structural measures such as voxel-based morphometry (VBM), although there are many functional studies about anger and its related elements in healthy young subjects. In a meta-analysis of positron emission tomography (PET) and functional magnetic resonance imaging (fMRI) studies about the functional anatomy of emotions, lateral orbitofrontal cortex (OFC) activity was reported in a higher proportion of studies targeting anger, relative to other emotions (Murphy et al. 2003), while anger induction was uniquely associated with increased regional cerebral blood flow in the right temporal pole and thalamus, as compared to a neutral condition using PET in healthy adults (Kimbrell et al. 1999). Trait Anger (T-Anger) was inversely associated with the strength of resting-state functional connectivity between the amygdala and contralateral middle OFC by resting-state fMRI in healthy subjects (Fulwiler et al. 2012). Anger was associated with activation of the left OFC, right anterior midcingulate cortex (aMCC) and bilateral anterior temporal poles in healthy men during PET (Dougherty et al. 1999). In a study using fMRI, in which healthy participants were insulted and then induced to ruminate about it, activity in the aMCC was positively correlated with self-reported feelings of anger and individual differences in general aggression (Denson et al. 2009). The aMCC is most prominently involved in cognitive control and decision-making (Vogt 2009), including conflict monitoring during attention (Botvinick 2007), target detection, response selection, set-shifting (Bissonette et al. 2013), and motivation (Bush 2010). Interestingly, instead of using insults, increased brain activity in happy lovers compared with unhappy lovers was seen in the aMCC using fMRI (Stoessel et al. 2011) and resting-state fMRI (Song et al. 2015). Furthermore, the aMCC strongly and reciprocally connects cognitive/attention and motor regions, including the dorsolateral prefrontal cortex (DLPFC), parietal cortex, and premotor cortex (PMC) (Bush 2010). The term dorsal ACC (dACC) is based only on a rough estimate from brain imaging studies (Procyk et al. 2016). Accordingly, use of a validated terminology is necessary, and a regional model by Vogt et al. (2003) is the standard (Procyk et al. 2016). The aMCC is often referred to as the dACC, but we use the term aMCC. As our research group reported previously (Takeuchi et al. 2012), structural imaging is particularly useful to investigate the anatomical correlates of a wide range of personal behaviors, because unlike fMRI studies, structural imaging findings are not limited to specific regions engaged in a task or the stimuli used during scanning. Furthermore, correlational studies using MRI techniques, including fMRI, to investigate the neural bases of individual differences have typically used established cognitive measures with proven reliability and validity scores (Canli et al. 2001; Gardini et al. 2009). However, brain structures associated with hostility outside of clinical human and animal studies have yet to be identified.

Based on the abovementioned findings, it was hypothesized that the nodes within the neural networks that underlie hostility involve widespread regions partly related to anger and the prediction of negative responses to future interpersonal interactions (Houston and Vavak 1991; Smith et al. 2004; Ramírez and Andreu 2006; Lemerise and Dodge 2008; Chen et al. 2012), including the aMCC. Thus, the primary purpose of the present study was to identify the gray matter (GM) structures within the neural networks that support the expression of hostility in healthy young adults. The present study used the hostile behaviors subscale (HBS) of the Coronary-prone Type Scale (CTS) for Japanese populations to assess hostility (Seto et al. 1997), and the associations of individual differences in hostility with regional gray matter density (rGMD) were evaluated using VBM (Good et al. 2001). Additionally, the present study investigated whether the rGMD associated with hostility was correlated with anger or with brain regions that have been previously implicated in the prediction of negative responses to future interpersonal interactions (Houston and Vavak 1991; Smith et al. 2004; Ramírez and Andreu 2006; Lemerise and Dodge 2008; Chen et al. 2012).

Moreover, males have been shown to exhibit a greater degree of hostility toward others more often than females in studies of university undergraduates (Ramirez et al. 2001) and of patients in psychiatric hospitals (Bruffaerts et al. 2004). Likewise, many studies have reported that domestically violent men have higher levels of anger and hostility than domestically nonviolent men (Eckhardt et al. 1997). Thus, the present study also investigated sex differences in hostility.

## Methods

### Subjects

The present study evaluated 777 healthy right-handed individuals (433 men and 344 women; mean age: 20.7 ± 1.8 years) as part of an ongoing project investigating associations among brain imaging, cognitive functions, aging, genetics, and daily habits (Takeuchi et al. 2010, 2011). The data derived from the present study will also be available for use by future studies investigating other themes. All subjects were university, college, or postgraduate students who had graduated from their respective institutions within 1 year of the initiation of the present experiment and who had normal vision. All university students undergo health examinations that include an assessment of their eyesight, but the eyesight of the study subjects was reassessed using an auto refractometer (Shin-Nippon ACCUREF 8001 Auto Refractometer, Ajinomoto Trading Inc.; Tokyo, Japan). During the recruitment process, all subjects were notified of the exclusion criteria, including the fact that those with mental and physical diseases could not participate in the experiment. The subjects were reminded of these criteria after the initial preliminary contact; thus, individuals who should have been excluded from the present study were eliminated before they came to the lab to participate. However, if a subject arrived to participate in the experiment and was previously excluded based on the stated criteria, they were asked to return home. It was not possible to determine how many potential subjects were excluded or dropped out during the various stages of the recruitment process because the study authors did not have access to the informal preliminary contacts and were not privy to the reasons why a particular subject was excluded. None of the subjects had a history of neurological or psychiatric illnesses and handedness was assessed using the Edinburgh Handedness Inventory (Oldfield 1971). Written informed consent was obtained from each subject prior to participation in the study in accordance with the Declaration of Helsinki (1991), and the study protocol was approved by the Ethics Committee of Tohoku University.

### Psychological outcome measures

#### Assessment of hostility

The CTS, which is a measure of Type A behavior patterns for Japanese individuals (Seto et al. 1997) that includes a HBS, was used to assess hostility in the present study. The HBS is based on the Hostile Aggression Inventory, which was derived from the Buss-Durkee Hostility-Guilt Inventory (propensity to assault, indirect hostility, irritability, negativism, resentment, suspicion, guilt, and verbal hostility) (Buss and Durkee 1957; Hata 1990). Type A behavior is an emotional syndrome characterized by a continuously harassing sense of temporal urgency and easily aroused hostility (Friedman et al. 1982). The CTS is a 30-item (HBS: 10-item) questionnaire that employs a six-point Likert scale response format ranging from “not true of me at all” (1) to “very true of me” (6); it yields a composite score of 10–60. This measure includes statements such as “I often quarrel” and “I am sarcastic or say evil things about some people in front of them.” The internal consistency of the CTS for normal subjects has a Cronbach’s *α* coefficient of 0.85 (Seto et al. 1997), and CTS scores are significantly and positively associated with scores on the Bortner scale, which has been validated and confirmed by structured interviews as an accurate measure of Type A behavior patterns (Wang et al. 2012). The CTS scores of patients with coronary heart disease are significantly higher than those of healthy subjects (Seto et al. 1997). Additionally, when the relationships of the CTS scores with social support and sex were examined in 213 male and 239 female Japanese college students, the CTS scores were inversely correlated with social support among both males and females separately (Sumi and Kanda 2001). There were no significant differences in the magnitudes of these coefficients between males and females.

#### Assessment of anger

The present study assessed anger using the State-Trait Anger Expression Inventory (STAXI), which is a self-report questionnaire consisting of 44 items and five subscales: State Anger, T-Anger, Anger-In, Anger-Out, and Anger-Control (Forgays et al. 1997). The STAXI has high internal consistency and high test–retest reliability in Asian populations (Bishop and Quah 1998). Because T-Anger and Anger-Out denote an outward direction of one’s anger (Angerer et al. 2000) and are thought to be related to hostility, the present study analyzed the relationships of the scores on these subscales with the identified brain regions.

### Psychometric measures of general intelligence

The present study used Raven’s Advanced Progressive Matrices (RAPM) to assess intelligence (Raven 1998) and to adjust for the effects of general intelligence on brain structures (Haier et al. 2004; Takeuchi et al. 2010). Each item in this measure consists of a 3 × 3 matrix with a missing piece that is completed by selecting the most appropriate of eight alternatives. The score for a subject on this test, which is the number of correct answers in 30 min, was used as a psychometric measure of individual intelligence in the present study.

### Behavioral data analyses

All behavioral data were analyzed with the IBM SPSS Statistics 22.0 software package (IBM Corp.; Armonk, NY). Sex differences in age and the scores on the cognitive measures (RAPM, HBS, T-Anger and Anger-Out) were analyzed with an analysis of variance (ANOVA), whereas Pearson correlation tests were used to evaluate relationships between HBS scores and scores on the T-Anger and Anger-Out subscales. A *P* value <0.05 corrected using the Bonferroni method was considered to indicate statistical significance.

### Image acquisition and analysis

#### Image acquisition

All MRI data were high-resolution T1-weighted structural images (T1WIs) acquired with a 3-T Philips Achieva scanner (Philips Medical Systems; Best, The Netherlands). All images were collected using a magnetization-prepared rapid gradient echo sequence with the following characteristics: 240 × 240 matrix, repetition time (TR) = 6.5 ms, echo time (TE) = 3 ms, field of view (FOV) = 24 cm, slices = 162, slice thickness = 1.0 mm.

#### Preprocessing of the T1WI data

All preprocessing of the structural data was performed with Statistical Parametric Mapping software (SPM8; Wellcome Department of Cognitive Neurology, London, UK) using new segmentation methods in SPM8 with the Diffeomorphic Anatomical Registration Through Exponentiated Lie algebra (DARTEL) registration process implemented in SPM8. Subsequently, all images were smoothed by convolving them with an isotropic Gaussian kernel of 8-mm full width at half maximum (FWHM; for a more detailed explanation, please see Supplemental Methods).

#### Statistical analyses

The present study investigated whether each rGMD was associated with individual differences in scores on the HBS, T-Anger, and Anger-Out. All statistical analyses of the morphological data were performed using SPM8, and only voxels that showed rGMD values >0.05 were included for each subject. The primary purpose for using GM thresholds was to define the periphery of the GM areas and to employ the smoothing process to effectively limit the areas that were to be analyzed to those likely to be GM. On the other hand, voxels outside these specified brain regions were more likely to be affected by signals outside the brain. By default, SPM8 masks the analysis of brain regions obtained by fMRI scans.

Threshold-free cluster enhancement (TFCE) with a family-wise error (FWE) correction was employed to define the cluster and to control for multiple comparisons (5000 permutations) (Smith and Nichols 2009), because the TFCE inference is fairly robust in response to the presence of non-stationarity in data (Salimi-Khorshidi et al. 2011).

#### Correlations between rGMD and hostility scores for all subjects

Multiple regression analyses were performed to analyze HBS scores as dependent covariates. The analyses were performed with sex, age, RAPM score, total intracranial volume [TIV; total GM volume + total white matter volume + total cerebrospinal fluid (CSF) volume], and T-Anger and Anger-Out scores as additional covariates. When total brain volume is included as a covariate in an analysis of density measures, the density of tissues that cannot be explained by total brain volume can be evaluated.

Correlations between rGMD and hostility scores for all subjects were assessed using TFCE with a FWE correction at a two-tailed significance level of *P* < 0.05.

#### Interaction effect of sex and scores on the HBS on rGMD

The present study also investigated whether the relationships between rGMD and HBS scores differed between sexes; in other words, we examined whether the interaction between sex and scores on the HBS affected rGMD. For each of the two whole-brain analyses, a voxel-wise analysis of covariance (ANCOVA) in which sex was a group factor (using the full factorial option of SPM8) was used. In one analysis, age, RAPM, T-Anger, Anger-Out, and HBS scores were used as covariates. Except for TIV, these covariates were modeled so that the unique relationship between each covariate and rGMD could be observed in each sex (using the Interactions option in SPM8); this allowed for the interaction effects of sex and the covariates to be investigated. The TIV covariate was modeled such that it had a common relationship with rGMD among both sexes. The interaction effects of sex and HBS score on rGMD were assessed using TFCE with FWE correction at a two-tailed significance level of *P* < 0.05.

#### Correlations between rGMD and T-Anger/Anger-Out scores for all subjects

Multiple regression analyses were performed using T-Anger or Anger-Out scores as dependent covariates. The analyses were performed using sex, age, RAPM score, and TIV as additional covariates. Correlations between rGMD and T-Anger or Anger-Out scores for all subjects were assessed using TFCE with FWE correction at a two-tailed significance level of *P* < 0.05.

#### Interaction effects of sex and T-Anger/Anger-Out scores on rGMD

The present study also investigated whether the relationships between rGMD and T-Anger or Anger-Out scores differed between sexes. The interaction effects of sex and HBS score on rGMD was assessed using TFCE with FWE correction at a two-tailed significance level of *P* < 0.05. For a more detailed explanation, please see Supplemental Methods.

#### Conjunction analyses for HBS and T-Anger/Anger-Out scores in the total sample

A conjunction analysis was performed analyzing the association of the HBS scores with T-Anger or Anger-Out scores using sex, age, RAPM score, and TIV as covariates. A *P* value <0.05 that was corrected at the non-isotropic adjusted cluster level and an underlying voxel significance level of *P* < 0.0025 were employed because conjunction analyses are the most statistically robust procedures that can be used to identify commonalities and differences between different data sets without interactional effects (Price and Friston 1997).

## Results

### Behavioral data

**Fig. 1:**
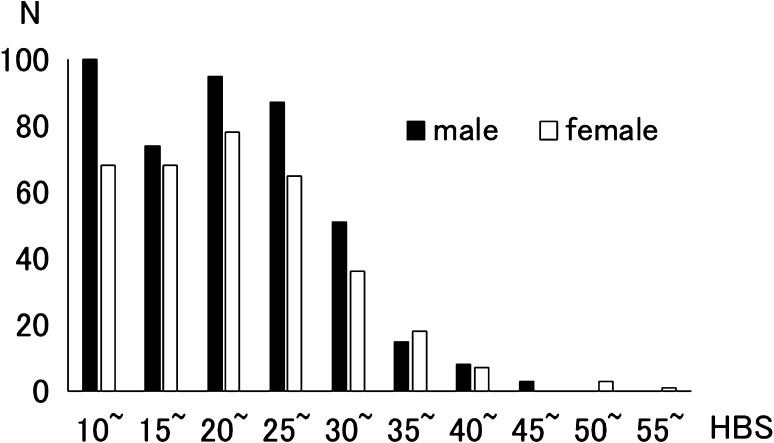
Distributions of HBS scores according to sex. Histogram separately displaying the distributions of scores of the hostile behaviors subscale (HBS) of the Coronary-prone Type Scale (CTS). Males: *filled square*, females *empty square*. *N* number of subjects

The distributions of the HBS scores for both sexes are shown in Fig. 1. Sex differences in age, scores on the RAPM, HBS, and T-Anger and Anger-Out scales, and ANOVA results for each sex are displayed in Table 1 (*P* < 0.05). HBS scores were significantly higher in males than females (ANOVA, *P* = 0.004). HBS scores were significantly positively correlated with those on T-Anger and Anger-Out (*P* < 0.001; Table 2).

**Table 1:** Sex differences in age; scores on the RAPM, HBS, and T-Anger and Anger-Out scales; and one-way ANOVA results

Measure	Total		Males (*N* = 433)		Females (*N* = 344)		*P*	*F*
	Mean	SD	Mean	SD	Mean	SD		
Age	20.7	1.8	20.8	2.0	20.6	1.7	0.099	2.7
RAPM	28.7	3.7	28.9	3.7	28.3	3.7	0.018*	5.6
HBS	22.2	8.3	23.0	8.3	21.3	8.1	0.004**	8.3
T-Anger	19.9	5.6	19.6	5.4	20.1	5.9	0.226	1.5
Anger-Out	18.0	4.3	18.0	4.4	17.9	4.2	0.923	0.009

*ANOVA* analysis of variance, *HBS* hostile behaviors subscale, *RAPM* Raven’s Advanced Progressive Matrices, *SD* standard deviation, *T-Anger* Trait-Anger

* *P* < 0.05, ** *P* < 0.001

**Table 2:** Pearson’s correlation tests for the HBS and STAXI scores

	HBS	Trait-Anger	Anger-Out
HBS	–		
Trait-Anger	0.647*	–	
Anger-Out	0.643*	0.574*	–

*HBS* hostile behaviors subscale, *STAXI* State-Trait Anger Expression Inventory

* *P* < 0.001, corrected with the Bonferroni method

### MRI data

#### Correlations between rGMD and hostility scores for all subjects

**Fig. 2:**
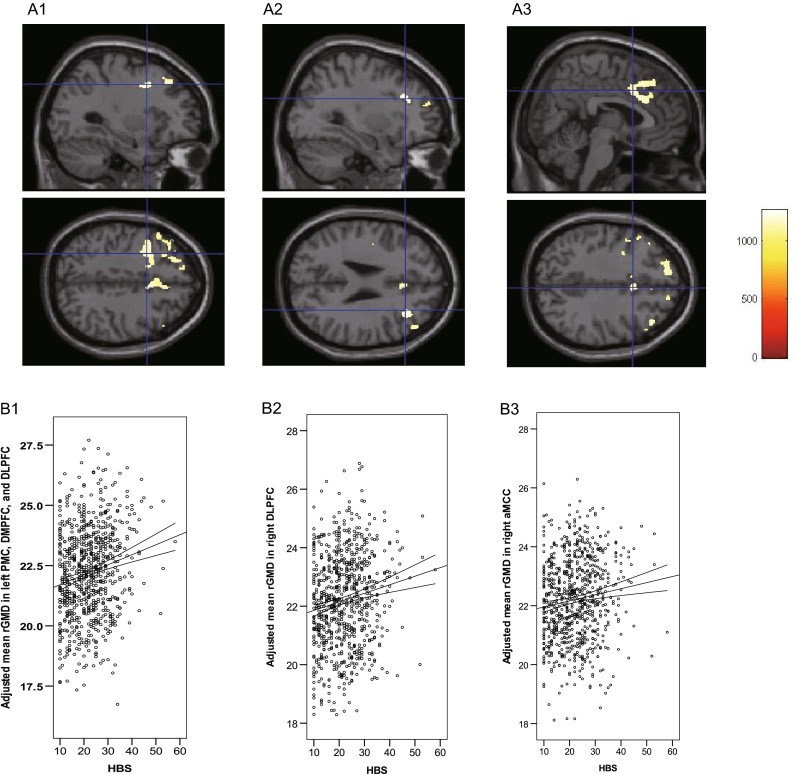
Brain regions exhibiting a correlation between mean rGMD and HBS scores. Multiple regression analyses were performed on the hostile behavior subscale (HBS) scores using sex, age, RAPM score, total intracranial volume [TIV; total gray matter (GM) volume + total white matter (WM) volume + total cerebrospinal fluid (CSF) volume], and Trait Anger (T-Anger) and Anger-Out scores as additional covariates. The *red-to-yellow color scale* indicates the *t* score of the positive correlation between the mean regional gray matter density (rGMD) values and the scores on the HBS [*P* < 0.05, two-tailed threshold-free cluster enhancement (TFCE) corrected with a family-wise error (FWE)]. Regions showing correlations were overlaid on a single T1-weighted image using the SPM8 toolbox. Areas with significant correlations included widespread regions mainly in the (**A1**) left frontal cortex from the left dorsomedial and dorsolateral prefrontal cortices (DMPFC/DLPFC), including the left premotor cortex (PMC), (**A2**) the right DLPFC, and (**A3**) anterior midcingulate cortex (aMCC). Residual plots with trend lines depicting the correlations between residuals in the multiple regression analyses with HBS scores as the dependent variable and other confounding factors as the independent variables; 95 % confidence intervals for the trend line are shown. The mean rGMD values for the significant clusters (**B1**) in the left PMC, DMPFC, and DLPFC; (**B2**) the right DLPFC; and (**B3**) the right aMCC

Multiple regression analyses were performed using HBS scores as dependent covariates. The analyses were performed using sex, age, RAPM score, and TIV as additional covariates. HBS scores were significantly positively correlated with rGMD in three anatomic clusters (Fig. 2; Table 3), which included the left DLPFC, dorsomedial PFC (DMPFC) and PMC (Fig. 2A1), the right DLPFC (Fig. 2A2), and the right aMCC (Fig. 2A3). The posterior OFC and limbic regions, except for the aMCC, were not included in the significant regions related to hostility. There were no significant negative correlations between rGMD and scores on the HBS.

**Table 3:** Brain regions exhibiting a significant correlation between rGMD and HBS score

Brain region	R/L	*x*	*y*	*z*	*TFCE*	Corrected *P* value (FWE)	Cluster size (*k*_E_)	*β*
PMC	L	−30	8	42	1195	0.010*	1580	0.173
DMPFC	L	−12	51	34	1182	0.011*		
DLPFC	L	−26	33	45	1157	0.013*		
DLPFC	R	32	27	27	1261	0.008*	435	0.132
DLPFC	R	42	33	34	1198	0.010*		
DLPFC	R	50	36	28	1160	0.012*		
aMCC	R	5	12	37	1186	0.011*	826	0.095
aMCC	R	5	21	30	1184	0.011*		
aMCC	R	8	6	49	1175	0.012*		

*DLPFC* dorsolateral prefrontal cortex, *DMPFC* dorsomedial prefrontal cortex, *FWE* family-wise errors, *HBS* hostile behaviors subscale, *aMCC* anterior midcingulate cortex, *L* left, *PMC* premotor cortex, *R* right, *rGMD* regional gray matter density, *TFCE* threshold-free cluster enhancement

* *P* < 0.05, two-tailed, FWE corrected

#### Interaction effect of sex and HBS scores on rGMD

No significant interaction effects of sex and HBS scores on rGMD were revealed by the ANCOVA using age, sex, TIV, and scores on the RAPM as covariates.

#### Correlations between rGMD and T-Anger/Anger-Out scores for all subjects

Multiple regression analyses were performed using the scores on T-Anger or Anger-Out as dependent covariates. The analyses were performed using sex, age, RAPM score, and TIV as additional covariates. No significant correlations were detected between rGMD and scores on the T-Anger or Anger-Out.

#### Interaction effect of sex and T-Anger/Anger-Out scores on rGMD

No significant interaction effect of sex or the T-Anger or Anger-Out score on rGMD was revealed by ANCOVA, using age, sex, RAPM scores, and TIV as covariates.

#### Conjunction analyses for HBS and T-Anger/Anger-Out scores for all subjects

Conjunction analyses were also performed to assess HBS and T-Anger or Anger-Out scores treating sex, age, RAPM scores, and TIV as covariates using a *P* value <0.05 that was corrected at the non-isotropic adjusted cluster level with an underlying voxel level of *P* < 0.0025. However, no significant correlation was observed between rGMD and the conjunction of HBS and T-Anger or Anger-Out scores.

## Discussion

To the best of our knowledge, this is the first study to investigate the brain regions that underlie hostility in a large sample at the whole-brain level. The primary finding is that the HBS scores of the subjects were significantly associated with higher rGMD values in the bilateral DLPFC, the right aMCC, and the left DMPFC and PMC. However, the conjunction analyses of the HBS and T-Anger or Anger-Out scores indicated that there was no significant overlap with rGMD. These findings are partly consistent with hypotheses suggesting that the neural nodes underlying hostility involve brain regions related to predictions regarding negative responses to future interpersonal interactions (Houston and Vavak 1991; Smith et al. 2004; Ramírez and Andreu 2006; Lemerise and Dodge 2008; Chen et al. 2012).

These brain structural outcomes confirmed that the regions implicated in functional studies of hostility are also associated with negative emotion and attitude. As mentioned in the Introduction, the aMCC region is related to various functions that are associated with hostility, especially conflict monitoring in attention (Parvaz et al. 2014), cognition (Hoffstaedter et al. 2014), emotion regulation (Kohn et al. 2014), and motor control (Hoffstaedter et al. 2014; Misra and Coombes 2015). Shackman et al. reported that negative affect and cognitive control are anatomically and functionally integrated in the aMCC (Shackman et al. 2011). The aMCC is enhanced by fear (surrogating measure; skin conductance) (Vogt et al. 2003). Interestingly, involvement of the aMCC during forgiveness, which is essentially the opposite of hostility, may reflect the homeostatic function of the decision-making processes that allow an individual to re-establish a subjective emotional balance following a hurtful interpersonal event (Ricciardi et al. 2013). Additionally, effective emotional regulatory behaviors, such as cognitive reappraisal and expressive suppression, are widely observed during healthy psychological adaptation, as evidenced by the fact that higher reappraisers report fewer negative emotions and more positive emotions (Kantor and Robertson 1977; Gross 2002). Accordingly, the aMCC plays critical roles in hostility, because this emotion is related to predicting negative responses to future interpersonal interactions (Houston and Vavak 1991; Smith et al. 2004; Ramírez and Andreu 2006; Lemerise and Dodge 2008; Chen et al. 2012).

It is important to explain the relationship between hostility and the PFC comprehensively. First, reappraisal and cognitive re-evaluation of a potentially emotionally arousing event seem to be based in top-down appraisal systems mediated by the PFC (Morawetz et al. 2015). In particular, the DLPFC is thought to be the central node of the prefrontal emotion regulation network (Morawetz et al. 2015). Interestingly, fMRI studies have revealed that reappraisal of high-intensity emotional responses is associated with increased activity in the left and right DLPFC, as well as in a more anterior portion of the DMPFC (Silvers et al. 2015). Accordingly, the DMPFC and DLPFC may be common non-specific neural nodes that support the experience of intense emotions, including hostility.

We should consider why the HBS and T-Anger and Anger-Out scores did not correlate with the higher rGMD, suggesting a weak association between hostility and the affected regions. First, hostility is also defined by negative cognitive appraisals of circumstances and individuals and represents a construct independent of the experience and expression of anger (Buss 1961). Moreover, hostility is a long-lasting emotion in humans, and expressing anger reduces anger, sometimes leading to a feeling of relief and satisfaction (Tyson 1998), whereas anger is a fleeting emotion that includes widespread negative emotions (Jackson 1972). That is, hostility seems to be different from anger itself. Previous studies have shown that the perception of anger triggers condition-specific activities in a wide set of brain regions, including the medial PMC (Pichon et al. 2009). An individual’s perception of the bodily expression of anger by another elicits activity in the medial PMC, which is thought to be important to prepare defensive behaviors (Grezes et al. 2013) and for external stimulus-driven actions and motor preparation (Pichon et al. 2012). Thus, it is reasonable to assume that the main function of the right lateral PMC during the expression of hostility is different from that of the neural correlates of T-Anger and Anger-Out (not lateral but medial PMC).

The present study has several limitations. First, as with previous studies from our lab using college student cohorts (Song et al. 2008; Jung et al. 2010; Takeuchi et al. 2010, 2011), only young healthy subjects with a high level of education were studied. The limited sampling of subjects with a full range of intellectual abilities is a common hazard when sampling from college cohorts (Jung et al. 2010), and it diminishes the ability to rule out the effects of age or educational level, which could strongly impact brain structures and influence the sensitivity of the analyses. Second, this study was cross-sectional, and therefore it could not determine the direction of causality among factors. Longitudinal cross-lag structural-equation analyses and experimental studies in humans have shown that hostility affects (and is affected by) social cognition and behavior. Last, educational status is linked to higher anger control (Boylan and Ryff 2013). Accordingly, the lack of a significant correlation between rGMD and the conjunction of HBS and Trait Anger/Anger-Out scores might be due to selection bias for highly educated young people in this study.

In conclusion, the present findings demonstrate that nodes within the neural networks underlying hostility include regions of the bilateral DLPFC, the left PMC and DMPFC, and the right aMCC. Additionally, the nodes within the neural networks include brain regions, particularly the aMCC, which have been previously implicated in negative predictions regarding negative responses to future interpersonal interactions. Further studies using more representative samples are needed to determine whether the present findings are generalizable across a wider range of populations.

## Electronic supplementary material

Below is the link to the electronic supplementary material.

Supplementary material 1 (DOC 55 kb)
