# Supplementary material for: The anterior midcingulate cortex as a neural node underlying hostility in young adults
Source: Brain Struct Funct. 2016 Feb 20;222(1):61–70. doi: 10.1007/s00429-016-1200-6 (PMC5225167; doi:10.1007/s00429-016-1200-6)
Supplement: Supplementary file 1 — Supplementary material 1 (DOC 55 kb) [file 429_2016_1200_MOESM1_ESM.doc]

**Supplemental Methods**.

***Preprocessing of the T1WI data***

As summarized by Takeuchi et al.(2012), the potential correlates of GM in VBM analyses may include the number and size of neurons and glial cells, the level of synaptic bulk, and the number of neurites (May and Gaser 2006; Takeuchi et al. 2011a). However, these correlates require histological confirmation. Various cognitive abilities have been associated with specific GM regions and neural structures, and the brain regions that support particular cognitive abilities or characteristics can therefore be directly investigated (Haier et al. 2004; Takeuchi et al. 2010). In this manner, structural imaging provides unique and distinctive information concerning the neural origin of individual cognitive characteristics. However, to the best of our knowledge, no studies have investigated the brain structures associated with hostility.

The preprocessing of structural data was performed using Statistical Parametric Mapping software (SPM8; Wellcome Department of Cognitive Neurology, London, UK) implemented in Matlab (Mathworks, Inc.; Natick, MA, USA). Using a new segmentation algorithm carried out in SPM8, the T1WIs of each individual were segmented into six tissues. For this process, the GM tissue probability map (TPM) was manipulated from maps implemented in the software so that the signal intensities of voxels with <0.25 became 0: (GM tissue probability of the default tissue GM TPM) + (white matter tissue probability of the default TPM). When a manipulated GM TPM is used, the dura mater is less likely to be classified as GM than when the default GM TPM is used, as substantial segmentation problems may occur.

In this new segmentation process, the default parameters were used, except that affine regularization was performed using the International Consortium for Brain Mapping template for East Asian brains. Then, the diffeomorphic anatomical registration through exponentiated lie algebra (DARTEL) registration process was implemented in SPM8 using the DARTEL import images of the six GM TPMs from the aforementioned new segmentation process. First, the template for the DARTEL procedure was created using imaging data from 63 subjects who had participated in a previous experiment in our laboratory (Takeuchi et al. 2011b). Next, the DARTEL procedure was performed on all data from the present study using the existing template and the default parameter settings. The resulting images were spatially normalized to the Montreal Neurological Institute (MNI) space to obtain images with 1.5 × 1.5 × 1.5 mm^3^ voxels.

***Interaction effect of sex and T-Anger/Anger-Out scores on rGMD***

For each of the two whole-brain analyses, a voxel-wise ANCOVA in which sex was a group factor was used. In one analysis, age, RAPM score, HBS score, and TIV were used as covariates. Except for TIV, these covariates were modeled so that the unique relationship between each covariate and rGMD could be observed in each sex; this allowed for the interaction effects of sex and the covariates to be investigated. The TIV covariate was modeled such that it had a common relationship with rGMD among both sexes. TFCE with a FWE correction was employed to define the cluster and the control for multiple comparisons (5000 permutations; Smith and Nichols, 2009). The interaction effect of sex and T-Anger or Anfger-Out score on rGMD was assessed using TFCE with a FWE correction at a significance level of *P <* 0.05, two-tailed.

**Supplemental References**

Haier RJ, Jung RE, Yeo RA, Head K, Alkire MT (2004) Structural brain variation and general intelligence. Neuroimage 23 (1):425-433. doi:10.1016/j.neuroimage.2004.04.025

May A, Gaser C (2006) Magnetic resonance-based morphometry: a window into structural plasticity of the brain. Current Opinion in Neurology 19 (4):407-411

Takeuchi H, Taki Y, Sassa Y, Hashizume H, Sekiguchi A, Fukushima A, Kawashima R (2010) Regional gray matter volume of dopaminergic system associate with creativity: evidence from voxel-based morphometry. Neuroimage 51 (2):578-585. doi: 10.1016/j.neuroimage.2010.02.078

Takeuchi H, Taki Y, Sassa Y, Hashizume H, Sekiguchi A, Fukushima A, Kawashima R (2011a) Regional gray matter density associated with emotional intelligence: evidence from voxel-based morphometry. Hum Brain Mapp 32 (9):1497-1510. doi:10.1002/hbm.21122

Takeuchi H, Taki Y, Hashizume H, Sassa Y, Nagase T, Nouchi R, Kawashima R (2011b) Failing to deactivate: the association between brain activity during a working memory task and creativity. Neuroimage 55 (2):681-687. doi: 10.1016/j.neuroimage.2010.11.052

Takeuchi H, Taki Y, Sassa Y, Hashizume H, Sekiguchi A, Nagase T, Nouchi R, Fukushima A, Kawashima R (2012) Regional gray and white matter volume associated with Stroop interference: evidence from voxel-based morphometry. Neuroimage 59 (3):2899-2907. doi: 10.1016/j.neuroimage.2011.09.064
